# Species Composition and Some Biological Features of Scorpions in Kazerun District, Southern Iran

**Published:** 2018-09-30

**Authors:** Mansour Nazari, Ali Najafi, Mohammad Reza Abai

**Affiliations:** 1 Department of Medical Entomology and Vector Control, School of Medicine, Hamadan University of Medical Sciences, Hamadan, Iran; 2 Department of Medical Entomology, School of Public Health, Tehran University of Medical Sciences, Tehran, Iran

**Keywords:** Scorpionida, Fauna, Kazerun, Iran

## Abstract

**Background:**

Scorpions have medical importance in the studied area with 2377 cases of envenoming during past six years. This study was the first to explore the scorpion species and dispersion in the Kazerun District during 2014–2015.

**Methods:**

The studied sites were selected based on different topographic conditions such as plain, foothill and mountainous which formed four geographical zones with three villages in each zone. The sampling was carried out twice each month throughout the year. Daytime collections were carried out using hand digging tools for moving stones and excavate the borrows, as well as night sampling, is done with the black light device. The coordinate of locations was recorded with a GPS. The collected specimens were maintained in 70% ethanol and identified using authorized keys.

**Results:**

Overall, 800 scorpions were sampled from different parts of Kazerun District, bringing the species richness to 9 belonged to 3 families of Buthidae, Scorpionidae, and Hemiscorpionidae. The *Mesobuthus eupeus* (84.6%) was prominent vice versa *M. caucasicus* (0.1%) had lowest abundance. Other species comprised *Compsobuthus matthiesseni* (5.3%), *Androctonus crassicauda* (5.0%), *Razianus zarudnyi* (2.0%), *Hemiscorpius lepturus* (1.5%), *Orthochirus* sp (0.9%), *Hottentotta zagrosensis* (0.4%), and *Scorpio maurus* (0.3%). The seasonal activity of the scorpions showed a lower peak in Mar, with the main peaks in Aug for the dominant species. *Mesobuthus caucasicus* is recorded for the first time in the Fars Province, southern Iran.

**Conclusion:**

All the known dangerous scorpions, including *H. lepturus*, *M. eupeus* and *A. crassicauda* were revealed in the studied area.

## Introduction

Considering ancient Iranian literature, as well as religious texts enlightened in the course of time, scorpion bites or scorpionism have been fully described which uncover the antiquity of this health problem in Iran. Information regarding scorpions in Iran is very limited, especially in Fars Province. The history of studies on the biology and ecology of scorpions is very rare in the country, and the majority of research is directed towards the descriptive studies of envenomation. A little information exists concerning scorpion bioecology and insufficient reports are available in different parts of the country.

Additionally, no robust scientific research have been discerned concerning scorpions presence in the various districts of Fars Province to determine their fauna and recognition of their habitats. Cases of scorpion sting in the country are an expression of the importance of this problem. The annual cases of scorpionism in Iran are projected from 40000–50000, resulting in 14–18 deaths per year (1). The dangerous consequences of the scorpion stings comprised the severe and lethal haemolysis, acute renal failure, deep necrotic wound, severe joint inflammation, temporary and permanent psychosis and death (2,3). The highest mortality due to scorpion stings is associated with *Hemiscorpius lepturus* Peters, 1861 in Iran, encountered as the most dangerous scorpion species in Khuzistan and Hormozgan provinces in southern Iran, where its abundance is high (4,5).

The scorpion fauna of Iran have immense richness due to the presence of different climatic condition consists 51 species (30 endemic to Iran) belonging to 18 genera and four families (6). However, due to population explosion in the cities and increasing urbanization, using of uncultivated land, and the construction of the buildings in the suburbs, led to the presence of the unwanted arthropods at indoor places. In addition, the establishment of rural homes or nomad tents are the ways which increase the possibility of scorpion entrance into the residential places. Scorpions occupy a wider range of habitats inside and outside buildings, in rural areas or suburbs of cities. The biodiversity of scorpions varies across different areas, hence their habitats also differ. Biological and environmental characteristics of scorpions according to their importance in health issues, and health risks caused by some species can provide practical and scientific solutions in dealing with them (7).

Recognition of scorpion habitats is the first step in combating and preventing scorpion sting in every area. In realization of scorpion habitats, the scorpions inhabit human hand-made shelters as well as natural hiding places (8). In this way, the method of elimination of breeding sites should be identified in order to control the scorpion population. Kazerun District is the second largest district in Fars Province, which has extensive plains and mountains with warm and dry climate, which provide a favorable condition for the scorpion activity. At present, 4 families, 18 genera, and 51 species have been reported, in which 7 species are venomous and dangerous to humans (6). The scorpion fauna of Fars Province have been studied, and 18 species reported. Of these, four species entailing *Compsobuthus persicus*, *M. eupeus persicus*, *Odontobuthus bidentatus* and *Scorpio maurus townsendi* were reported for the first time in Fars Province (9). As the scorpionism is one of the important health problems, however, the study on scorpion fauna has the poorer history in the Kazerun.

This study was conducted to reveal the fauna, abundance and some biological feature of the scorpions at selected localities during 2014–2015.

## Materials and Methods

### Study area

The Kazerun District is located in the southwestern slope of the Zagros Mountain chain, Fars Province at 51°39′15″E and 29° 37′10″N, with an elevation less than 860m above sea level covering an area of nearly 4119km^2^. It is bordered by Shiraz to the east, Norabad to the north, Bushehr to the west and southwest, and Farashband to the southeast. Kazerun comprises diverse climates, such that there is a mountainous cold zone with an elevation of 3000m in the north, and a warm and dry zone in the south with an elevation below 2000m. Water is traditionally supplied by “Qanat” traditional irrigation system, wells, springs, and rivers, in addition to rain. Kazerun is also renowned pertaining to the presence of narcissus meadows and Parishan Lake. In Kazerun, a variety of agricultural products are cultivated, including wheat, barley, rice, lentils, beans, tobacco, wild almonds, sesame, citrus fruits, and dates.

### Site selection

**Fig. 1.**
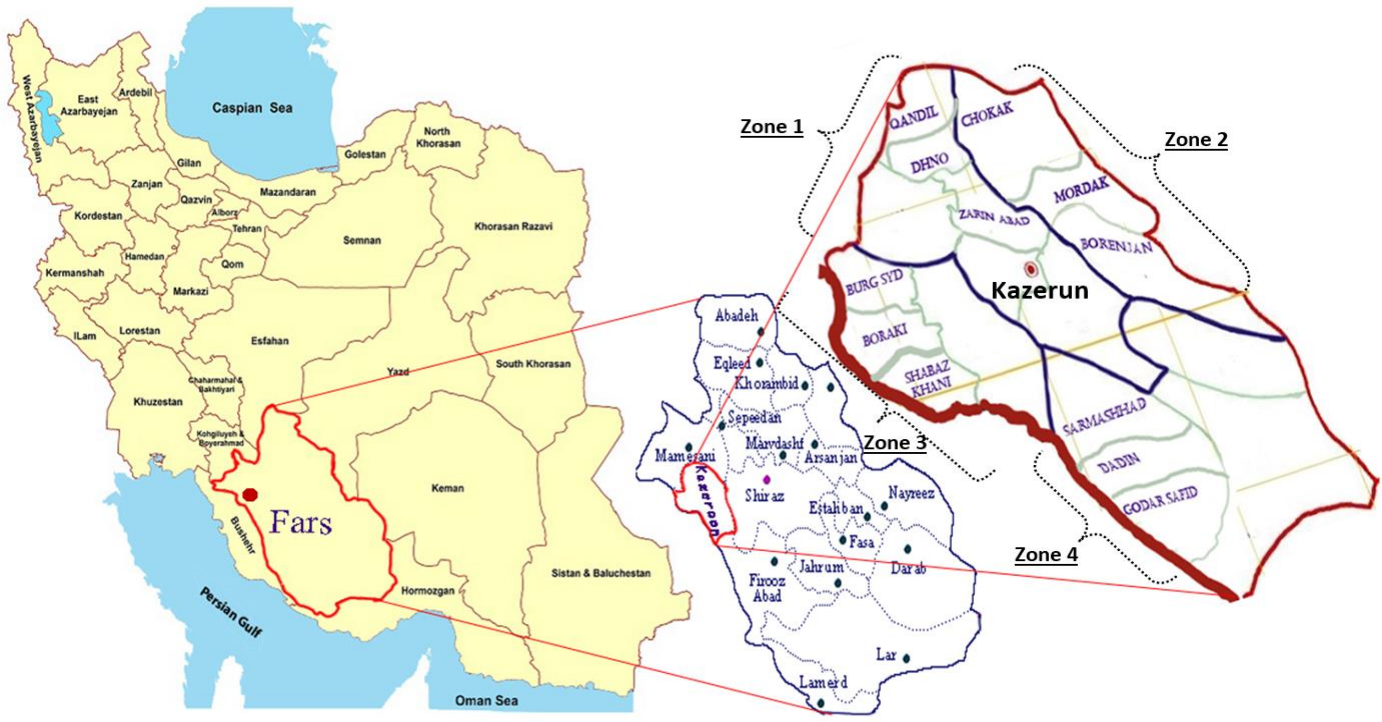
The selected zones in Kazerun district and their geographical positions in the Fars Province and the country

For site selection, the district was divided into 4 zones, and for each zone, 3 topographic conditions were considered as plain, foothill and mountainous. The climatic features of each zone are as follows:

**Zone 1:** Located in the north west of Kazerun District (Fig. 1), where the selected villages included Zarinabad (plain), 51°36′33″E, 29°39′11″N, at 835m above sea level (ALV) with mean of annual temperature (MAT) 23 °C and mean of annual relative humidity (ARH) 30%, Dehno Enghelab (mountainous/MAN), 51°34′45″E, 29°47′26″N, 987m (ALV), 30 °C (MAT), 46% (MRH), and Qandil (MAN), 51°34′46″E, 29°53′00.4″N, 926m (ALV), 29 °C (MAT), 35% (MRH), at a distance of 10, 20 and 35km respectively from the central part of the district where the ground is rocky with various low hills surrounding the villages.
**Zone 2:** Located in the north-eastern of Kazerun District (Fig. 1), where the selected villages included Burenjan (MAN), 51°52′24″E, 29°35′28″N, 1334m, 20 °C (MAT), 28% (MRH), Chekak (MAN), 51°39′54″E, 29°52′34″N, 1174m (ALV), 32 °C (MAT), 37% (MRH), Murdak (MAN), 51°48′36″E, 29°38′38″N, 1173m (ALV), 32 °C (MAT), 38% (MRH), at a distance of 30, 45 and 65 km respectively from the central part of the district with alluvial rocks and covered by oak trees.
**Zone 3:** Located in the west of Kazerun District (Fig. 1), where the selected villages included Buraki (plain/PLN) 51°20′28″E, 29°33′44″N, 498m, 34 °C (MAT), 45% (MRH), Borje Seyed (PLN), 51°22′33″E, 29°35′41″N, 504m (ALV), 32 °C (MAT), 43% (MRH), Shahbaz Khani (PLN), 51°19′58″E, 29°36′42″N, 494m (ALV), 36 °C (MAT), 46% (MRH), at a distance of 45, 60 and 65km respectively from the central part of the district where there are many palm gardens.
**Zone 4:** Located in the southwest of Kazerun District (Fig. 1), where the selected villages included Dadine Olya (foothill/FOT) 51°52′06″E, 29°19′04.5″N, 646m, 30 °C (MAT), 45% (MRH), Ghodar Sefid (FOT), 51°43′46″E, 29°35′41″N, 808m (ALV), 28 °C (MAT), 43% (MRH), Sarmashjad (FOT), 51°19′58″E, 29°36′42″N, 815m (ALV), 25 °C (MAT), 40% (MRH), at a distance of 45, 50 and 70km respectively from the central part where agriculture activities are carried out, and some uncultivated lands are covered of wild shrubs.

### Scorpion samplings

The sampling was carried out twice each month throughout the year during 2014–2015. The scorpions were actively sought both at daytime by using hand digging tools for turning the stones, digging the borrows and collecting the scorpions by long forceps as well as portable ultraviolet devices used at night (2). A portable Garmin™ GPS device was used for recording the geographical coordinates of the collection localities in the field. A variety of possible habitats were examined both at indoor and outdoor e.g. animal shelters, yards, beneath stones, within rock crevices, beneath trunk of fallen trees, trashes and dry stone walls partitioning agricultural fields and gardens. The courtyards of rural residence were sampled after sprinkling water on the grounds at night, which elicited the attraction of the scorpions. Moreover, an attempt was made to capturing the burrowing scorpions, in which disperse colonies were seen around the selected zones. The collected scorpions were maintained in ethanol (70%) using long forceps and were eventually labeled. All the specimens were identified using authentic scorpion keys (10–15) were deposited at the Museum of Department of Medical Entomology, Hamadan University of Medical Sciences.

## Results

### Species composition

**Fig. 2.**
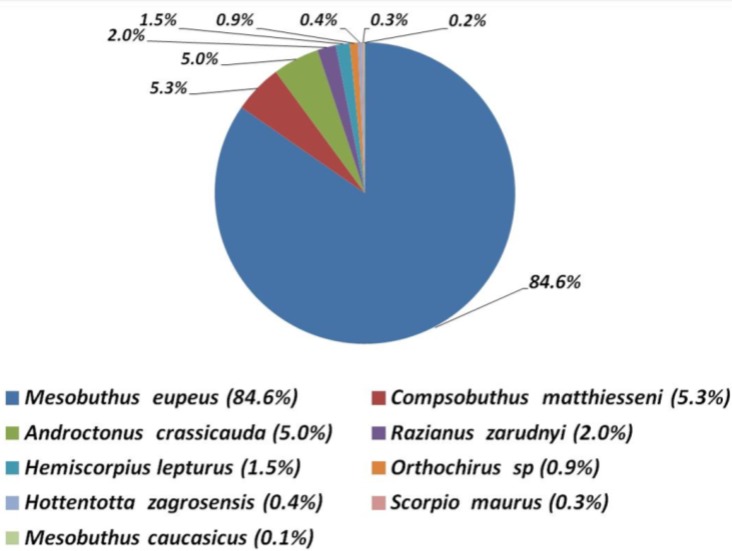
Species composition of the scorpions found in Kazerun District, Fars Province, southern Iran 2014–2015

Overall, 800 scorpions were collected from the 4 selected zones, which includes three families of Buthidae (98.3%), Scorpionidae (1.4%) and Hemiscorpionidae (0.3%), and 8 genera and species were identified with different abundance (Fig. 2). *Mesobuthus eupeus* has the highest density in the studied area (84.4%) whereas, M*. caucasicus* (0.1%) showed the lowest density. The scorpion species were categorized according to low, moderate and high densities in the studied area.

#### Bioecological features of the species *Mesobuthus eupeus*:

**Fig. 3.**
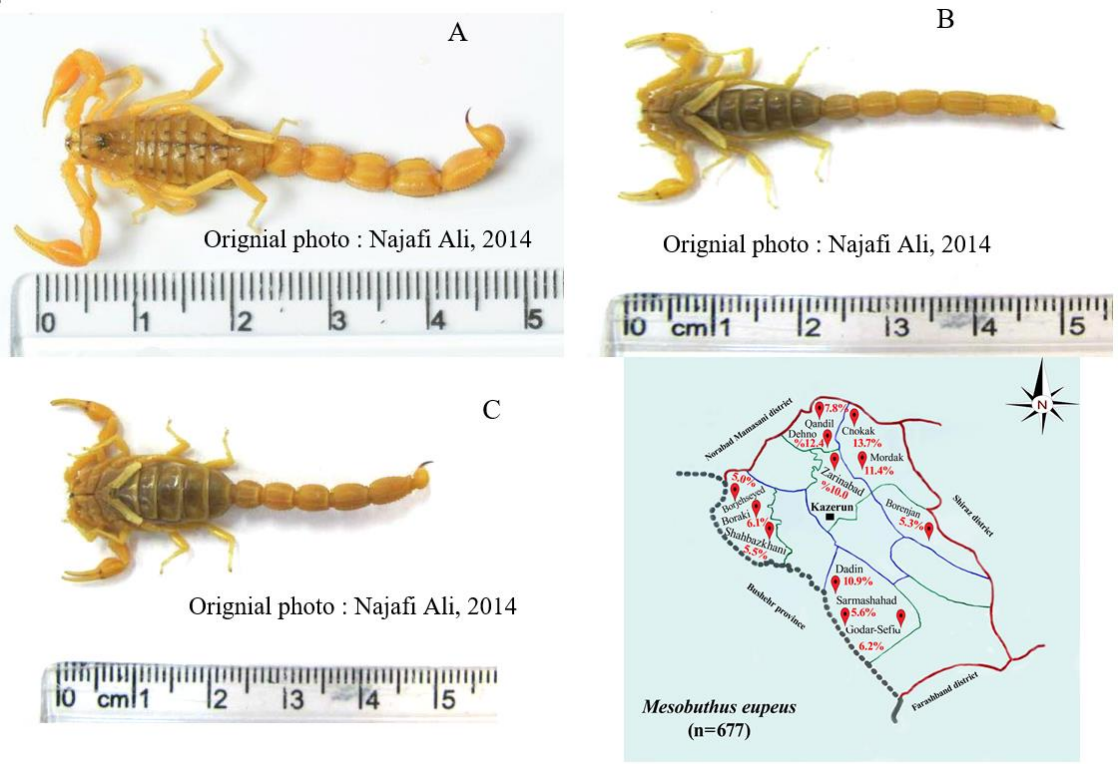
Ventral (A) and dorsal (B) views of male and female (C) of *Mesobuthus eupeus*. The map shows its dispersion and abundance at the studied zones, Kazerun District, Fars Province, 2014–2015 (original photos)

**Fig. 4.**
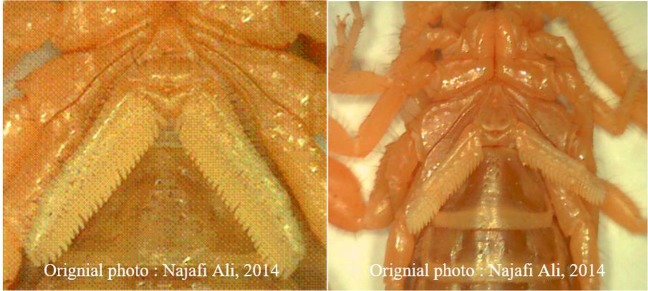
Ventral view of *Mesobuthus eupeus* in male (left) with 22 to 28 pectinal teeth compared to female (right) with 16–23 one, Kazerun District, Fars Province during 2014–2015 (original photos)

**Fig. 5.**
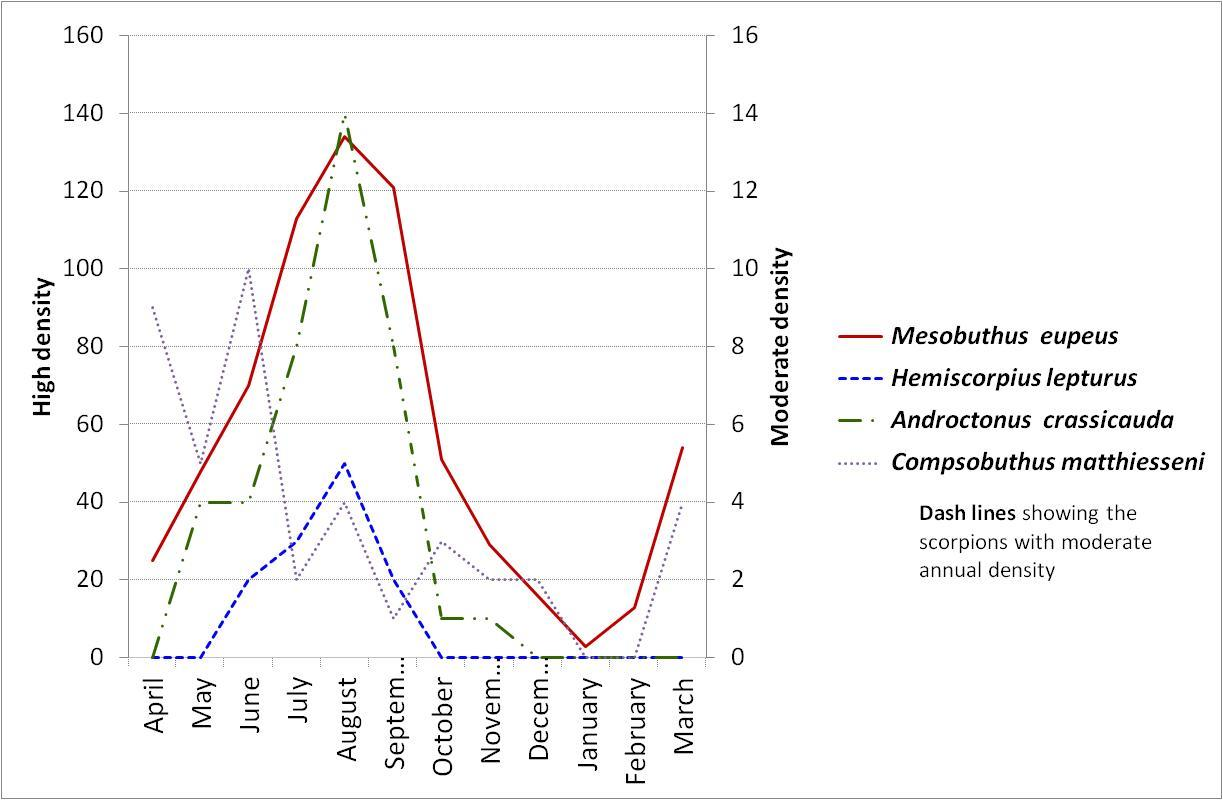
Monthly fluctuation of prevalent species found in Kazerun District, southern Iran 2014–2015

A well-known species belonging to the family Buthidae, and consisting of 84.6% of the total collected specimens, with a male-to-female sex ratio of 207:470 (Fig. 3), were collected in Kazerun district where it showed a universal dispersion in all the studied zones with different topographic conditions e.g. plains and mountainous parts (Fig. 4). This scorpion was discovered in places of different elevations ranging between 499 and 1225 meters above the sea (Table 1). The extensive distribution of this species in different zones due to climatic variations is necessary for determining the biological conditions of the scorpion. The monthly activity of this species extended throughout the entire months of the year, beginning of Apr ending in Mar, with a noticeable peak in Aug (Fig. 5). *Mesobuthus eupeus* were recognized as burrowing scorpion especially around the shaded parts of stone humps arranged between the farms as well as this species found underneath the rocks and dumped walls.

**Table 1.** Biodiversity of scorpions and the geographical features of selected zones in Kazerun District, Fars Province, southern Iran 2014–2015

**Geographical Zone and mean of Elevation above sea level**	**Direction of the zone in the district and Coordinates**	**Selected villages and Mean of annual temperature and RH**	**Species richness and No. of species**	**Species composition**								

				***M. eupeus***	***H. lepturus***	***A. crassicauda***	***S. maurus***	***C. matthiesseni***	***H. zagrosensis***	***R. zarudnyi***	***M. caucasicus***	***Orthochirus* sp**
Zone 1 (**916m**)	Northwest **29°46′12.5″N; 51°35′41.3″E**	Dehno-Zarrinabad-Gandil **27.3°, 37.0%**	0.7 (**6**)	205 (**30.3%**)	6 (**50.0%**)	12 (**30.0%**)	0 (**0.0%**)	18 (**42.9%**)	2 (**66.9%**)	16 (**100%**)	0 (**0.0%**)	0 (**0.0%**)
						Zone 2 (**1227m**)	Northeast **29°42′33.3″N; 51°42′38.0″E**	Borenjan-Chekak-Mordak **28.0°, 34.0%**	0.7 (**6**)	206 (**30.4%**)	6 (**50.0%**)	11 (**27.5%**)	2 (**100%**)	6 (**14.3%**)	0 (**0.0%**)	0 (**0.0%**)	0 (**0.0%**)	3 (**42.9%**)
Zone 3 (**499m**)	West **29°35′42.3″N; 51°20′39.7″E**	Boraki-Borjesheid-Shahbazkhani **34.0°, 45.0%**	0.3 (**3**)	112 (**16.5%**)	0 (**0.0%**)	15 (**37.5%**)	0 (**0.0%**)	0 (**0.0%**)	0 (**0.0%**)	0 (**0.0%**)	0 (**0.0%**)	4 (**57.1%**)
Zone 4 (**756m**)	South west **29°17′11.8″N, 51°45′19.0″E**	Dadin Olya-Ghodarsefid-Sarmashhad **27.7°, 43.0%**	0.6 (**5**)	154 (**22.7%**)	0 (**0.0%**)	2 (**5.0%**)	0 (**0.0%**)	18 (**42.9%**)	1 (**33.3%**)	0 (**0.0%**)	1 (**100%**)	0 (**0.0%**)
Total	4 zones	**12** villages	**9** species	677 (**84.6%**)	12 (**1.5%**)	40 (**5.0%**)	2 (**0.3%**)	42 (**5.3%**)	3 (**0.4%**)	16 (**2.0%**)	1 (**0.1%**)	7 (**0.9%**)

#### Hemiscorpius lepturus:

**Fig. 6.**
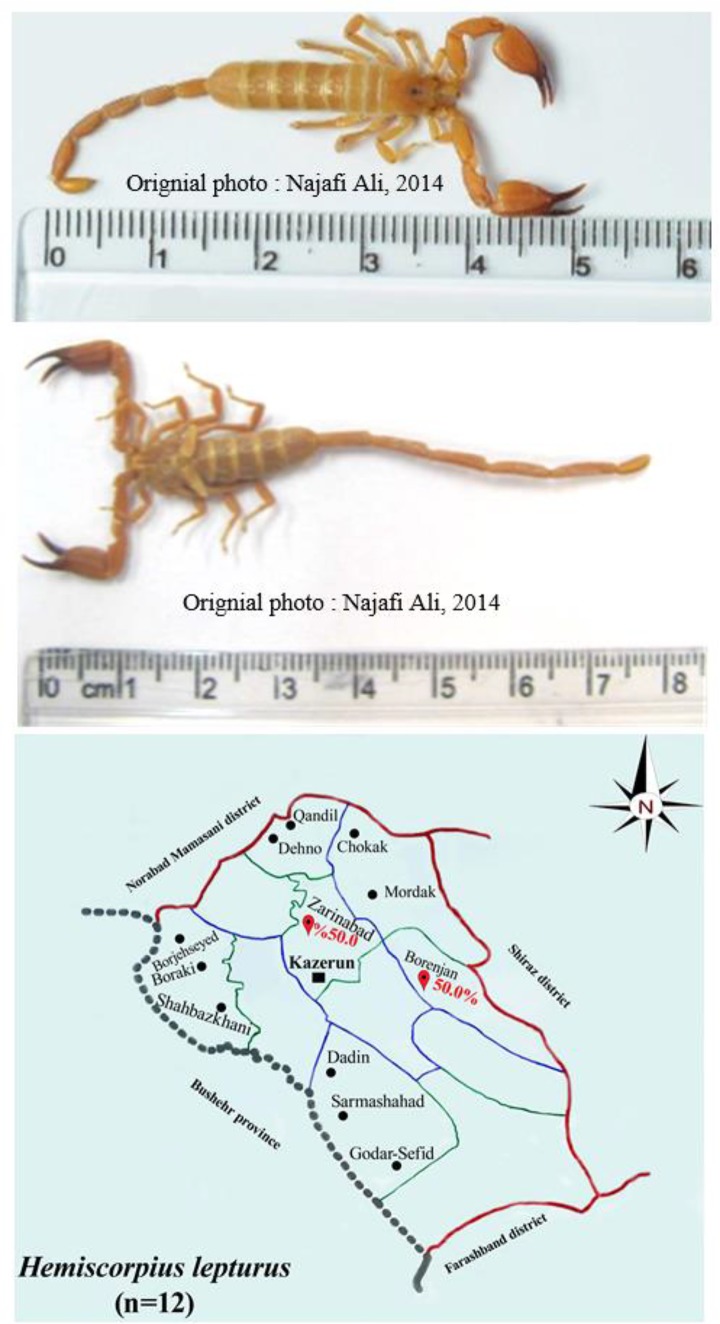
Ventral and dorsal views of female (up) and male (down) of *Hemiscorpius lepturus*. The map shows its dispersion and abundance at studied zones, Kazerun District, Fars Province, 2014–2015 (original photo)

Belongs to the family Hemiscorpiidae and locally named as ″Gadim″ in southern Iran, and consisting of 1.5% of the total scorpions with a male-to-female sex ratio of 2:10 (Fig. 6), were collected in the Kazerun district. This species was only found in Zones 1 and 2 where is mountainous with the elevation range 916–1227 meters above sea level (Table 1), and an equal density of 50% each zone. The monthly activity of this species was limited to the warmer months of the years, beginning of July ending in Sep, characterized by a peak in Aug (Fig. 5). The hiding places of *H. lepturus* were revealed under the stones and rocks.

#### Androctonus crassicauda:

**Fig. 7.**
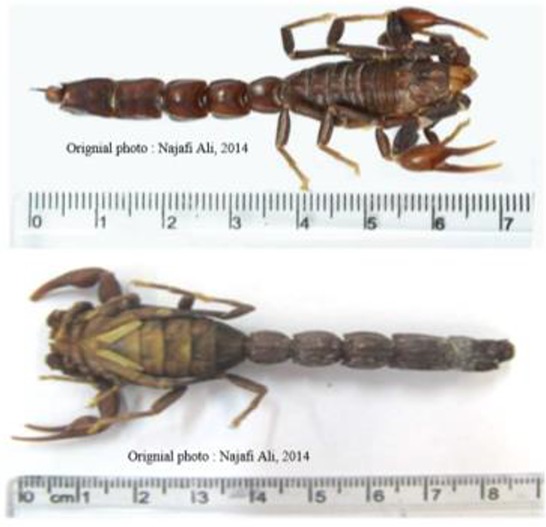
Ventral (down) and dorsal (up) views of female *Androctonus crassicauda*, Kazerun District, Fars Province, 2014–2015 (original photos)

**Fig. 8.**
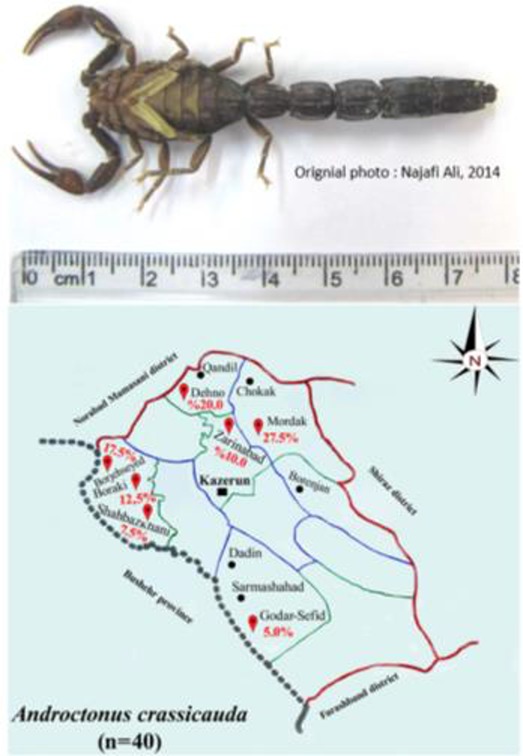
Ventral view of male *Androctonus crassicauda*. The map shows its dispersion and abundance at the studied zones, Kazerun District, Fars Province, 2014–2015 (original photo)

Or fat-tailed scorpion belongs to the family Buthidae, which consisted of 5.0% of all sampled scorpions in Kazerun District with a male-to-female sex ratio of 4:36 (Fig. 7). This scorpion was established in all the studied zones with the nearly equal occurrence (Fig. 8). The monthly activity of this species started from May and ended in Nov, projecting a peak in Aug (Fig. 5). The habitats of *A. crassicauda* were realized in both indoors (under the dry humped trees, ground crevices, crevices of mud walls, broken bricks of the walls), and outdoors (under the humped bricks and stones, under loose stones and ant holes). It was easily attracted to vehicular lights.

#### Scorpio maurus:

**Fig. 9.**
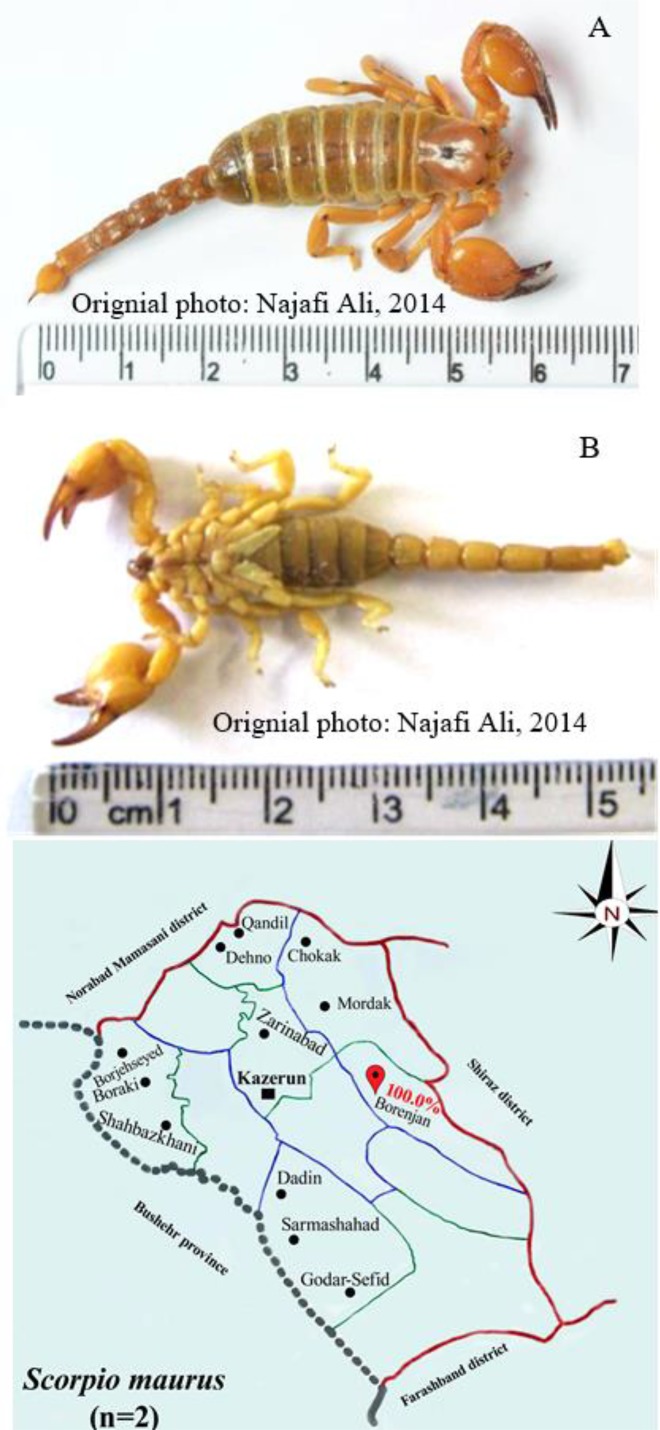
Dorsal (A) and ventral (B) of female of *Scorpio maurus*. The map shows its dispersion and abundance in the zone 2, Kazerun District, Fars Province, 2014–2015 (original photos)

**Fig. 10.**
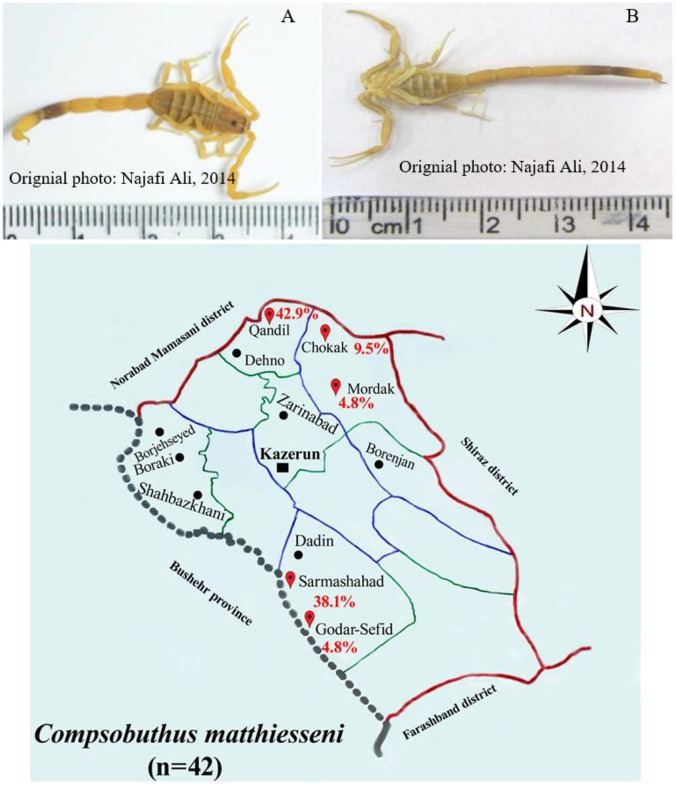
Dorsal (A) and ventral (B) of female *Compsobuthus matthiesseni*. The map shows its dispersion and abundance at the studied zones, Kazerun District, Fars Province (2014–2015) (original photos)

Belongs to the family Scorpionidae which only two females was found (Fig. 9) in low density (0.3%) in the September (Fig. 10) at higher elevation (1227m), which covered by the *Quercus* trees. This scorpion is a burrow-living species, having various deep holes (up to 45cm) and found in the wheat farm at the zone 2.

#### Compsobuthus matthiesseni:

Belongs to the family Buthidae found in moderate density (5.3%) (Fig. 10) with a male-to-female sex ratio of 5:37 sampled in Kazerun District. This scorpion was present in all the studied zones with nearly equal occurrence, with the exception of Zone 3 where the lower elevation has. The monthly activity of this species started from Apr and ended in Mar with a peak in Jun (Fig. 10). The evidence shows the adaptation of this species to temperate conditions than to higher temperatures. The sampled scorpions were collected from rock crevices and bark of trees, where having the high humidity.

#### Hottentotta zagrosensis:

**Fig. 11.**
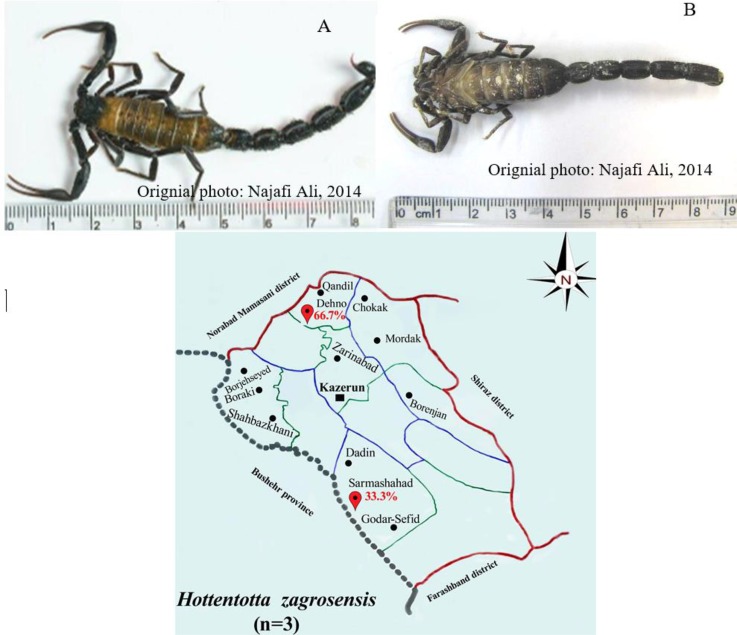
Dorsal (A) and ventral (B) of female *Hottentotta zagrosensis*. The map shows its dispersion and abundance at the specified zones, Kazerun District, Fars Province, 2014–2015 (original photos)

Belongs to family Buthidae which only three females were found in low density (0.3%) (Fig. 11), with an annual occurrence in July and Aug (Fig. 10) in the Kazerun district reported from Zones 1 and 4 where the elevation ranged between 756–916 meter above sea level. The collected specimens were found around the stones in semi-arid parts.

#### Razianus zarudnyi:

**Fig. 12.**
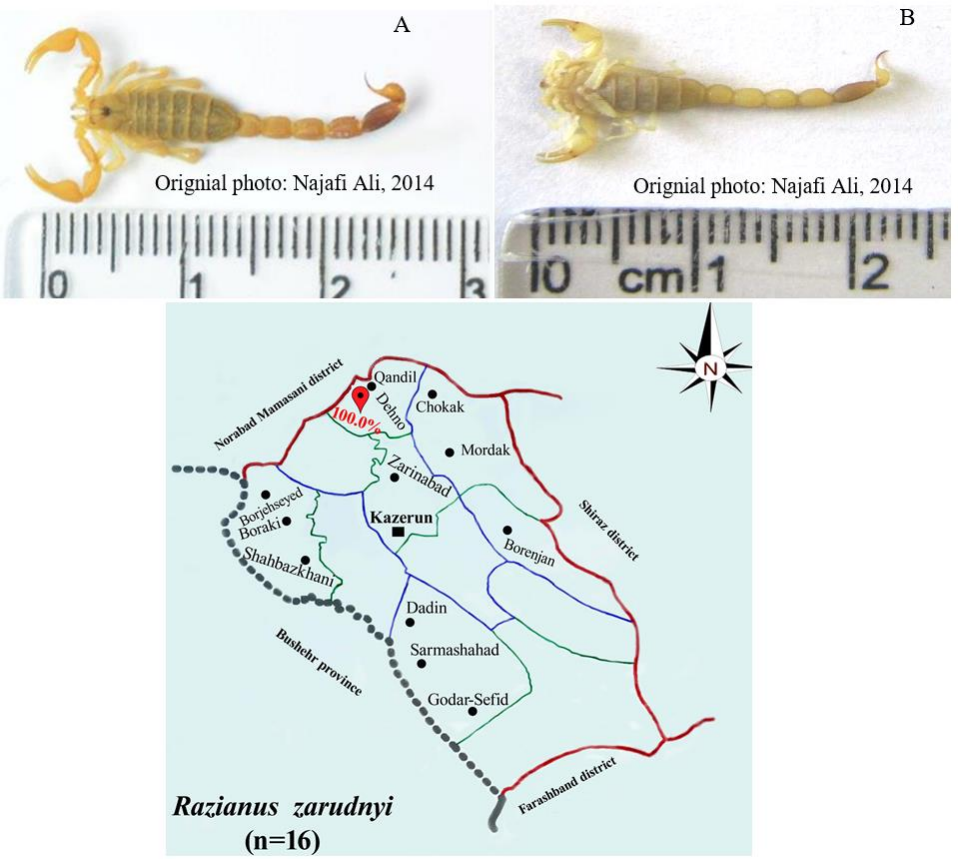
Dorsal (A) and ventral (B) of male of *Razianus zarudnyi*. The map shows its dispersion and abundance at the zone 1, Kazerun District, Fars Province (2014–2015) (original photos)

Belongs to Buthidae which consisted of 2.0% of all scorpions sampled in Kazerun district (Table 1) with a male-to-female sex ratio of 4:12 (Fig. 12). This scorpion was only evident in zone 1, in the northeast of Kazerun, where the elevation was 916 meters above sea level. The monthly activity of this species started from Mar and ended in Sep with a peak in Mar. The more success in collection of this species ensued from Jul to Sep (Fig. 10), whereas the collection was zero during the other months, with the exception of March which showed a peak. The hiding places of this species were in rock crevices.

#### Mesobuthus caucasicus:

**Fig. 13.**
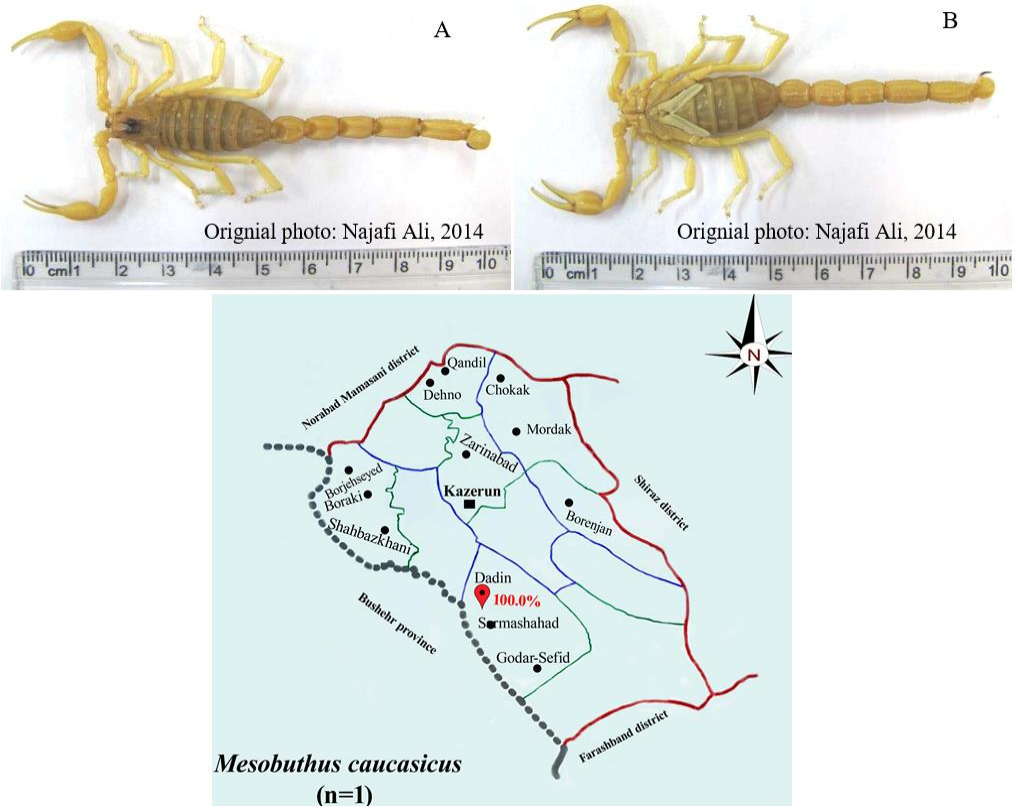
Dorsal (A) and ventral (B) of female *Mesobuthus caucasicus*. The map shows its dispersion and abundance at the specified zone 4, Kazerun District, Fars Province (2014–2015) (original photos)

Belongs to the family Buthidae which was only one female found in low density (0.1%) (Fig. 13) with their occurrence in Jul (Fig. 10), present in Zone 4 where the elevation was 499 meters above sea level. These species was found under stones and lumps.

#### Orthochirus sp.

**Fig. 14.**
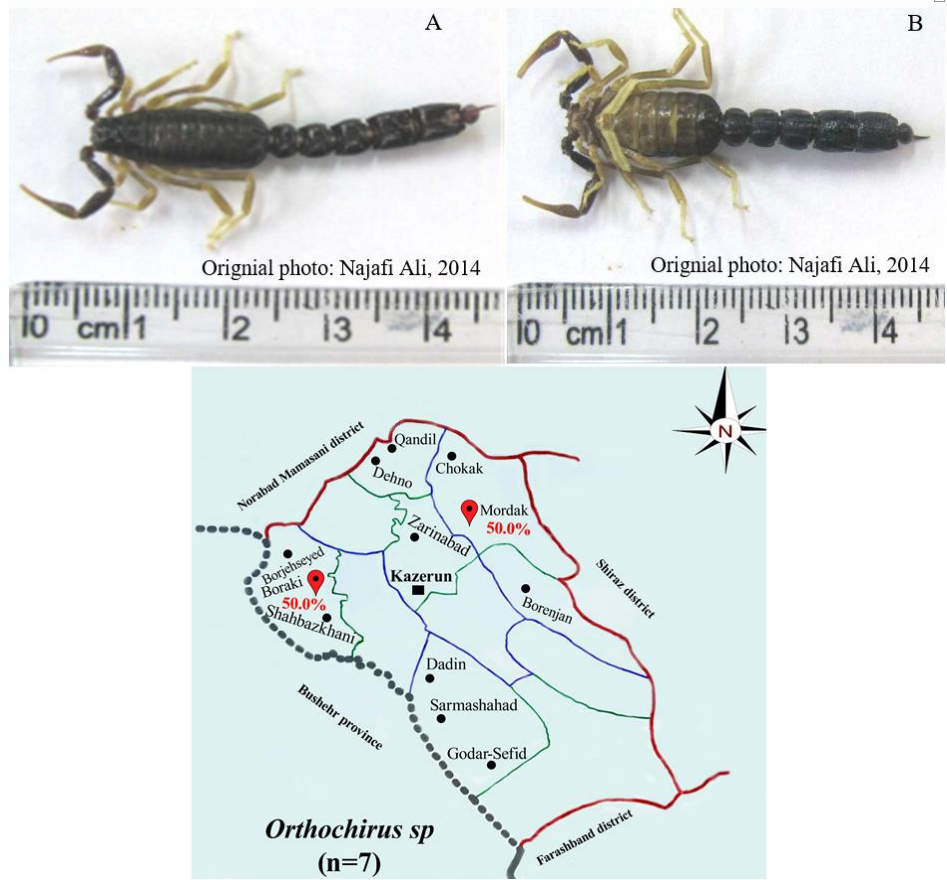
Dorsal (A) and ventral (B) of male *Orthochirus* sp*.* The map shows its dispersion and abundance in the zone 4, Kazerun District, Fars Province, 2014–2015 (original photos)

Belongs to family Buthidae found in low density (0.9%) (Fig. 14), with a male-to-female sex ratio of 1:6 and occurred during Jun to Jul (Fig. 10) in Zone 4, where the elevation ranged 499–1227 meter above sea level. This species was as rapid moving scorpions on the ground and active at night and found around the dry rubble stones near the foothills with low vegetation.

### Seasonal activity

The monthly activity of different species, including those with low, moderate and high density, was recorded. In the spring, the activity of most species of scorpions was minimal, but it was improved due to the occurrence of higher temperatures in the habitats during summer. The peak of activity of most species was in Aug (Fig. 5), whereas the *C. matthiesseni* showed a moderate density (5.3%) in Jun–Jul (Fig. 10).

## Discussion

In spite of the public health importance of scorpionism Kazerun District (16) and its considerable richness of scorpions, limited studies have been directed on the faunistic and biodiversity of these arthropods. The scorpion envenomation has the most public health importance in Kazerun District, however, the current study is the first trial in determining the scorpion fauna in the area. The collected scorpion species comprised *M. eupeus*, *H. lepturus*, *A. crassicauda*, *S. maurus*, *C. matthiesseni*, *H. zagrosensis*, *R. zarudnyi*, *M. caucasicus,* and *Orthochirus* sp. The most dangerous scorpion, *H. lepturus* was ranked in second from the point of view of density. Dispersion of this species was affected by the type of soil and its humidity, topographic condition and climate. This species was only revealed in the zones 1 and 2, where the topographic conditions are mountainous. A majority of the human victims were stung by *H. lepturus* (16), while a few more were stung by *A. crassicauda*, but in over half of the cases the latter species was not known in southern and southwestern parts of Iran (4).

The scorpion fauna of Kazerun District was compared to those of adjacent provinces and districts. The predominant species, *M. eupeus* had similar ranking in both Shiraz District (17) and Kohgiluyeh and Boyer-Ahmad Province (18, 19). During a comprehensive study on scorpion fauna in Fars Province, 18 species were recorded belongs to three families whereby 15 species originated from Buthidae, 1 species from Scorpioniidae and 2 species from Hemiscorpioniidae (9). Fifty percent of the recorded species in Fars Province were discovered in Kazerun District. Two other comprehensive studies regarding scorpion fauna were carried out during 2008–2011 in the Kerman Province, in the eastern border of Fars Province, which showed 8 to 13 species, belonging to the families of Buthidae and Hemiscorpioniidae, respectively (19, 20). Seven out of nine species disclosed in this study were included in the checklist of Bu shehr Province, which reported 15 species (21). Moreover, a faunistic study was done in the Khuzistan Province in the west of Kazerun District (22). Surprisingly, the lowest similarity (28.6%) was observed between the fauna of Bushehr Province and Kazerun District. The most common species were *A. crassicauda*, *M. eupeus phillipsii*, *S. maurus townsendi* and *R. zarudnyi*. The latter finding can lead the scorpionologist towards the detecting the biological factors involved. For the first time, *M. caucasicus* which explored from Zone 4 at 499m above sea level. The latter species had not mentioned in the published checklist of Fars Province (9).

*Orthochirus* sp. which found in Zone 2 and 3 needs more taxonomic works for precise identification. Parameters such as topography, climate, texture, and humidity of soil were effective parameters. Careful planning and designing of a comprehensive database could be essential in assisting of the primary health system in the reduction of scorpion sting and its related complications.

## Conclusion

This study revealed the presence of 9 species belonging to 3 families Scorpionidae (2 species), Buthidae (5 species) and Hemiscorpiidae (1 species). The most dangerous scorpion *H. lepturus*, which known as ″Gadim″ in its local name is revealed in Zone 1 and Zone 2 where considering as a highland in Kazerun district.
